# Scanning Electron Microscopy versus Transmission Electron Microscopy for Material Characterization: A Comparative Study on High-Strength Steels

**DOI:** 10.1155/2021/5511618

**Published:** 2021-05-04

**Authors:** Nicolas Brodusch, Salim V. Brahimi, Evelin Barbosa De Melo, Jun Song, Stephen Yue, Nicolas Piché, Raynald Gauvin

**Affiliations:** ^1^McGill Electron Microscopy Research Group, Department of Mining and Materials Engineering, McGill University, Montréal, Québec, Canada H3A 0C5; ^2^McGill Hydrogen Embrittlement Facility, Department of Mining and Materials Engineering, McGill University, Montréal, Québec, Canada H3A 0C5; ^3^Object Research Systems, 760 St-Paul West, Suite 101, Montreal, Quebec, Canada H3C 1M4

## Abstract

The microstructures of quenched and tempered steels have been traditionally explored by transmission electron microscopy (TEM) rather than scanning electron microscopy (SEM) since TEM offers the high resolution necessary to image the structural details that control the mechanical properties. However, scanning electron microscopes, apart from providing larger area coverage, are commonly available and cheaper to purchase and operate compared to TEM and have evolved considerably in terms of resolution. This work presents detailed comparison of the microstructure characterization of quenched and tempered high-strength steels with TEM and SEM electron channeling contrast techniques. For both techniques, similar conclusions were made in terms of large-scale distribution of martensite lath and plates and nanoscale observation of nanotwins and dislocation structures. These observations were completed with electron backscatter diffraction to assess the martensite size distribution and the retained austenite area fraction. Precipitation was characterized using secondary imaging in the SEM, and a deep learning method was used for image segmentation. In this way, carbide size, shape, and distribution were quantitatively measured down to a few nanometers and compared well with the TEM-based measurements. These encouraging results are intended to help the material science community develop characterization techniques at lower cost and higher statistical significance.

## 1. Introduction

Historically, electron microscopy techniques are widely used to characterize microstructures corresponding to various alloying and fabrication conditions. Scanning electron microscopy (SEM) allows one to obtain characterization and distribution of the multiphase components of an alloy at the macro- and microscales, i.e., from microns to centimeters. However, its performance at finer scales is highly dependent on the gun technology available with the microscope. In particular, a SEM fitted with a thermionic emitter is limited to microscale resolution while field emission [1] provides larger brightness and smaller probe dimensions and thus higher resolution at the nanoscale [2]. Traditionally, for fine microstructure characterization, the transmission electron microscope (TEM) has been preferred since it provides atomic scale resolution with various imaging modes and crystallographic information using in situ electron diffraction techniques [3]. Unfortunately, TEM requires long and sophisticated sample preparation techniques [4, 5] in order to obtain an electron transparent sample. The most used techniques are focused ion beam cutting [6, 7], jet electropolishing [8], or broad beam milling [9]. Consequently, TEM and its necessary specimen preparation techniques thus suffer from expensive user fees in many electron microscopy centers. More importantly, the most dramatic drawback of TEM is, without a doubt, its limited areal coverage. Since the electron transparent areas produced by the preparation techniques cited above are at best of a few tens or hundreds of square micrometers, the microstructure of metals and alloys deduced from TEM characterization must be interpreted with care. Note that this might also hold for SEM close to its resolution limit in some cases. In comparison, SEM examines very large area, up to several hundreds of square centimeters in most microscopes and requires moderate preparation times in general, and the SEM user charges are more affordable for research and industrial laboratories. In other words, during the time required to prepare and analyze a TEM metallic alloy lamella, SEM can provide microstructural information from several samples for cheaper costs. However, a combination of SEM and TEM is the best for assessing the microstructure of an alloy in order to image large areas and nanoscale details.

The recent generation of field-emission SEMs (FE-SEM) provides now the necessary spatial resolution to probe the surface at the nanoscale [10] and more particularly those fitted with cold-field emitters (CFE-SEM) [11]. Since they provide the highest brightness, large probe current densities can be achieved and, when combined to efficient electron detectors, high signal-to-noise ratio (SNR) images with various contrasts can be obtained [12].

In this paper, we report on advances in the characterization of high-strength steels in a high-resolution CFE-SEM. This category of steels exhibits fine martensitic microstructures and a complex carbide precipitation and distribution at the micro- and nanoscales. A comparison of the main features of the microstructure for four different alloys is provided using electron diffraction and surface imaging capabilities of the SEM. A method based on deep learning tools [13] is presented to characterize the carbide distribution to take full account of the high representativeness of the SEM analysis combined with the CFE-SEM high-resolution imaging capabilities. A similar technique was previously reported for segmenting SEM images of ultrahigh carbon steels [14], but in the present study, we report the use of deep neural networks for segmenting very fine precipitates at the nanometer scale.

## 2. Materials and Methods

### 2.1. Materials

Four specimens were produced from two high-strength steels, AISI 5140SKV (A2) and 4135MLV (A6). Their weight percent carbon equivalent content [15] was very similar, namely, 0.64% and 0.67% for alloys A2 and A6, respectively. Their chemical compositions are given in Table 1. Each alloy was heat treated to achieve an HRC Rockwell hardness near 35 and 50, and the heat treatment history for each specimen is given in Table 2 as well as the true measured hardness. Samples M12 and M16 were produced with alloy A2 while M23 and M26 were produced with alloy A6. Based on the hardness values obtained as well as on the additional fourth tempering stage, samples M12 and M26 will be qualified as “quasi as-quenched” while samples M16 and M23 will be qualified as “tempered.”

### 2.2. Sample Preparation

TEM characterization was performed on 3 mm discs cut and punched from specimen sheets ground down to 100 *μ*m with silicon carbide papers with grits from 400 to 1200. Electron transparency was achieved using electropolishing using a solution consisting of 10 vol.% perchloric acid in methanol at around -40°C with an electropolishing voltage of 16 V. For SEM, the samples were cut in squares of approximately 1 × 1 cm^2^ and ground similarly to the TEM sheets. Further polishing was conducted with diamond suspensions of 3 *μ*m and 1 *μ*m particle size. The obtained flat surfaces were then finally polished 10 minutes with a mixture of colloidal silica and hydrogen peroxide (30 vol.%) to accelerate material removal in a ratio of 1/1. All polishing materials were from ANAMET, Boucherville, Canada. Each sample surface was then submitted to a cleaning step using an ozone cleaner (ZoneSEM, Hitachi High-Technologies, Rexdale, Canada) for 30 minutes and a pressure value of 40 (arbitrary units of pressure of the instrument).

### 2.3. Electron Microscopy Instrumentation

TEM investigations were conducted at the Canadian Centre for Microscopy (CCM) at McMaster University, Hamilton, ON, Canada, with a Philips CM12 (Thermo Fisher, Waltham, MA, USA) transmission electron microscope operating at 120 kV in bright-field (BF) and dark-field (DF) imaging modes. All SEM images were obtained with either the SU-8230 or the SU-9000EA Hitachi CFE-SEMs (Hitachi High-Technologies, Rexdale, Canada) located at the Mining and Materials Engineering Department at McGill University, Montréal, QC, Canada. Electron channeling contrast (ECC) images were obtained using a semiconductor photodiode-type backscatter electron (BSE) detector (PD-BSE) located below the microscope pole-piece normal to the beam direction. The working distance was 7-8 mm, and the distance between the BSE detector and the specimen surface was 2-3 mm. The secondary electron (SE) images were collected simultaneously with the ECC images with a single scan with the in-lens upper detector (upper) of the microscope. Both ECCI and SE imaging were conducted with a 10 kV accelerating voltage. Energy-dispersive spectroscopy was done using a Bruker Quantax FlatQuad spectrometer attached to the Hitachi SU-8230 CFE-SEM with an accelerating voltage of 4 kV. This voltage was chosen to reduce the volume of emission of X-rays in order to reduce the spatial resolution of the EDS analysis which was required to target the nanometer size carbide precipitates. In addition, this voltage was selected to allow an optimum overvoltage for all elements present in the alloy.

### 2.4. Carbide Dimensions and Martensite Lath Width Measurements with TEM

The size distribution of martensite lath width was obtained by measuring manually the width on a line normal to the direction of the grain long axis on several TEM-BF images (see Figure S1 in the supplementary material file). Carbide dimensions were obtained by manually measuring each particle length and width on the BF images from several regions. Each histogram was composed of several hundreds of measurements. The ImageJ software [16] was used to obtain the intensity profiles from the TEM-BF images.

### 2.5. Electron Backscatter Diffraction (EBSD)

SEM provides various automatic feature measurements on a crystalline specimen using EBSD. This electron diffraction technique is, of course, slower compared to the time required to acquire TEM images, and typical acquisition time is on an hour scale for a 1000 × 1000-pixel image. However, recent advances in the EBSD camera technology [17, 18] allows now reducing the EBSD map acquisition time to just a few tens of minutes for large images. All EBSD data were acquired with a Bruker e-Flash HD detector attached to the Hitachi SU-8230 CFE-SEM controlled by the Quantax/Esprit software 2.1.0. The accelerating voltage was 15 kV, and the probe current was around 5 nA. The acquisition step size was 33, 64, 38, and 38 nm for samples M12, M16, M23, and M26, respectively.

The phases used for indexing were austenite fcc with space group 225 ($Fm\bar{3}m$, *a*, *b*, *c* = 3.66 Å) and martensite bct with space group 139 (I 4/mmm, *a*, *b* = 2.847 Å, *c* = 3.018 Å) both implemented in the Esprit software. The martensite lattice parameters were extracted from the Joint Committee on Powder Diffraction Standards (JCPDS) PDF card #00-044-1293. The *c*/*a* ratio of the martensite structure used here was larger than what was expected from previous studies for low carbon steels [19, 20] and corresponds to a carbon content of 1.35% wt. It must be inferred that none of these studies reported the effect of alloying element fractions as well as the impact of carbon precipitation on their relation between tetragonality and the total carbon content. In addition, the SU-8230 CFE-SEM used to produce the EBSD maps produces a strong magnetic field affecting a large volume of the specimen chamber below the microscope pole piece. This magnetic field deflects some of the forward and backscattered electrons emitted towards the camera, and thus, the Kikuchi bands, as seen by the detector, tend to appear slightly bent. The resulting angles measured between pairs of bands are slightly affected, resulting in larger errors in the indexing procedure. We indexed ten high-quality EBSD patterns obtained on sample M23 with phases containing increasing carbon content, from 0.0 to 2.0% wt., with and without the objective lens magnetic field. This resulted in the patterns being acquired without the magnetic field being best indexed with phases containing between 0.6 and 0.7 ± 0.3% wt. of carbon while it was found to be between 1.0 and 1.1 ± 0.6% wt. when the objective lens field was applied. For this reason, we considered the choice of this phase still relevant as it provided better indexing compared to the true bct structure corresponding to the alloys investigated in this work.

The EBSD maps reported in this work were presented in their original form, i.e., raw data without smoothing postprocessing. To produce grain maps, the pixels of each map were gathered together as a function of pixel orientations to compute grain maps where the pixels of each grain are assigned the same color. For each map, the grain distribution was extracted using the Esprit software, and only grains with more than ten pixels and grain boundaries larger than 15° were considered to produce the grain maps.

### 2.6. Electron Channeling Contrast Imaging

Similarly to the diffraction contrast obtained in TEM, SEM can provide crystallographic imaging, and this is known as electron channeling contrast imaging (ECCI) [21, 22]. In TEM bright-field (BF) imaging, the pixel intensity depends on the fraction of primary electrons diffracted out of the bright-field collection cone via the objective aperture. The amplitude of the intensity loss is driven by the combined effect of the amplitude (diffraction) and mass-thickness (atomic number dependent) contrasts [3]. Similarly, in the SEM, the BSE fraction emitted from the sample is modulated by the crystal orientation [23] and the material mean atomic number (*Z*) [2] from the emission volume where those BSEs were produced. Thus, the variation in crystal orientations and distributions induced by the material processing are reflected in the ECC images in an identical fashion as that observed in the bright-field TEM images. It must be mentioned here that also dislocations can be detected since these induce local plane bending, thus producing local changes of orientation. Crystal defect imaging has been reported in several materials and alloys [24–29] and is now recognized as a viable imaging technique.

### 2.7. Carbide Imaging with Secondary Electrons

Carbide characterization is essential to understand the behavior of high-strength steels under servicing conditions. Therefore, it is of primary importance to analyze their chemistry, size and shape, and distribution in the material under investigation. TEM, through the mass-thickness contrast, provides a certain amount of contrast between the steel matrix and the carbide precipitates. However, due to the strong diffraction contrast in bright-field imaging, carbide visibility is highly dependent on the grain orientation. Since BSE imaging is also sensitive to *Z*, carbides generate a contrast in the BSE image but are also affected by the orientation contrast produced by ECC. In fact, the mean atomic number of the carbides is so close to that of the matrix that the variations of the BSE coefficient due to channeling, which can be as high as a few percent [21], have the effect of increasing or decreasing the BSE intensity from the matrix [30]. Thus, the precipitate contrast is reversed depending if the parent grain is or is not in channeling condition, rendering the detection of those precipitates very difficult.

**Figure 1:**
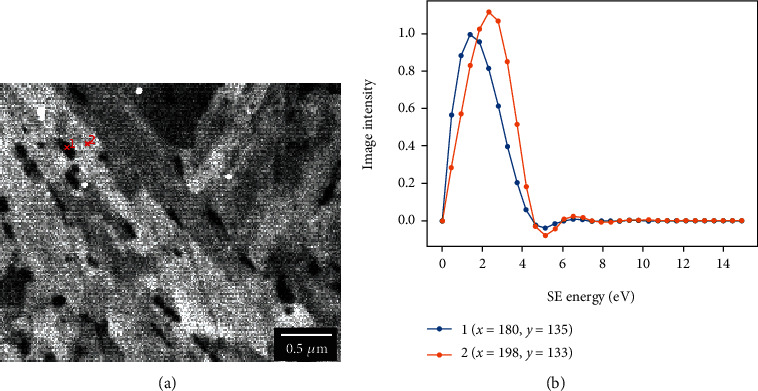
Secondary electron image (a) and secondary electron energy distributions (b) from a carbide (1) and from the matrix (2). The profiles were obtained by collecting the electron signal with the SU-8230 upper detector similarly to Hashimoto et al. [37].

To circumvent this issue, SE imaging with the in-lens detector of the CFE-SEMs was used to provide high contrast between the carbides and the matrix. The chemistry of the carbides was analyzed by energy-dispersive spectroscopy (EDS) mapping. Characteristic EDS images for Fe and C are shown in Figure S2 as well as the corresponding SE image. This contrast can be explained by a combination of emission characteristics of two secondary electrons. It has been reported that the SE yield from oxides and, more generally, from insulators, was systematically greater than yields obtained for metals [31, 32]. Considering the final sample polishing step using a mixture of colloidal silica suspension with hydrogen peroxide, it is expected that a native oxide layer forms at the surface of the iron-rich matrix phase [33]. Consequently, it is assumed that the oxidized matrix releases a larger number of SEs compared to the carbides. This effect, combined with the large fraction of the SE1 type of secondary electrons (produced by the primary electrons) captured by the in-lens detector, which are highly affected by the compounds' electronic structure via the dielectric function [34, 35], might explain the significant contrast observed on the SE images. Recently, Liu and coworkers [36] reported high carbide contrast with SEs in a ferritic 9Cr-1Mo steel after introducing XeF_2_ in the observation chamber of a dual-beam-focused ion beam microscope. The authors did not give the mechanism that led to the contrast, but it is assumed that a similar surface modification of the matrix took place, increasing in that way its SE emission yield. This hypothesis was verified by collecting the energy profile from a carbide particle (1—blue line) and its neighboring matrix (2—orange line). The profiles were obtained using the filtering capability of the SU-8230 upper detector in a similar manner as reported by Hashimoto and coworkers [37] and are shown in Figure 1.  Figure 1(a) shows a typical SE image obtained by the upper detector when collecting the full energy spectrum of secondary electrons, and the secondary electron energy distribution profiles are given in Figure 1(b). The profile intensity corresponds to the image intensity extracted from the set of filtered images. From the profiles in Figure 1(b), it is clear that carbides generate fewer secondary electrons than the iron matrix and that they have lower exit energies and, in the end, less SE intensity, thus validating our approach for imaging carbide distribution in the samples.

### 2.8. Carbide Segmentation with Deep Learning

Each SEM image provided hundreds of particles, and it was unrealistic to manually obtain quantitative measurements. Since these images were showing high and uniform contrast over full image fields, it was decided to apply artificial intelligence models to segment and measure particle size and distributions via a deep learning approach. A UNet multilayer neural network [38] was generated and trained with a few images (3 to 5) for each sample. Each image was manually mouse segmented. To reduce the time required for manual segmentation necessary to feed the network with training output, only a cropped portion of each image was used for the training. In fact, we found that multiple cropped areas were more beneficial for the model efficiency than one single image, assuming the same total time for manual segmentation. This permitted SEM acquisition parameter variations, such as noise, magnification, beam astigmatism, and martensitic grain background, from one image to another, to be accounted for. Once the manual segmentation output results were fed into the neural network, the training time was about 10 to 20 minutes per sample and the segmentation using the model was applied to the set of images, which comprised between 10 and 20 images for each sample.

The deep learning model training process and segmentation were done using the Dragonfly software (ORS, Object Research System, Montreal, Canada). The computer used was a 64-bit operating system with an Intel(R) Core(TM) i9-9900K CPU @ 3.60 GHz, 3600 MHz with 8 cores and 16 logical processors, 64 GB physical RAM memory, and a NVIDIA Quadro P2000 graphic card. The stopping criteria used to terminate the model training was the default stopping criteria implemented in the deep learning tool in Dragonfly. To improve the robustness of the deep neural network, the Dragonfly deep learning tool was set to include the variation of several parameters like image brightness, contrast, noise, scale, rotation, or shear in the training protocol. Each segmented image was then submitted to size and shape measurements using the same software where the Python (http://www.python.org) scikit-image library [39] was implemented. Carbide size and shape measurements were carried out with the “regionprops” method from this library.

## 3. Results

### 3.1. Comparison of the Microstructure Components

#### 3.1.1. Microstructure Overview

**Figure 2:**
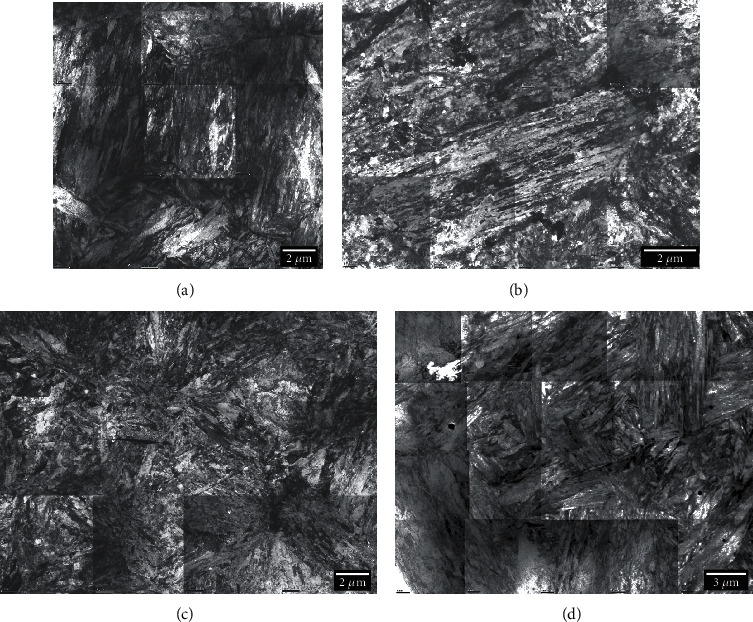
Microstructure overview for samples M12 (a), M16 (b), M23 (c), and M26 (d) as seen using bright-field TEM. Each image is resulting from a montage using several single images permitting to adjust the brightness and contrast of the field of view of each image. Due to the varying thickness over the image field of view, the bright-field contrast is dominated not only by diffraction but dramatically by the mass-thickness contrast mechanism.

The microstructure obtained using the TEM in a bright-field imaging mode for the four alloys is displayed in Figure 2. Each image is a montage of several single BF-TEM images obtained at positions where the specimen thickness was varying. In fact, this procedure is essential to produce a final image with limited mass-thickness variations due to local thickness variations. These variations were introduced by the specimen preparation technique used to produce electron transparent areas around the central hole. This also allows a reduction of the contribution of the objective projection lens spherical aberration [3] to the final montage, since only higher-magnification images were captured for this purpose. This is seriously inconvenient since it requires a large amount of data in addition to the processing and acquisition times necessary to record and process the set of images. Even with this treatment, the local variations are still visible and prevent a clear view of the microstructure. However, the martensitic microstructure as well as the carbide distribution in space can still be assessed from these images.

**Figure 3:**
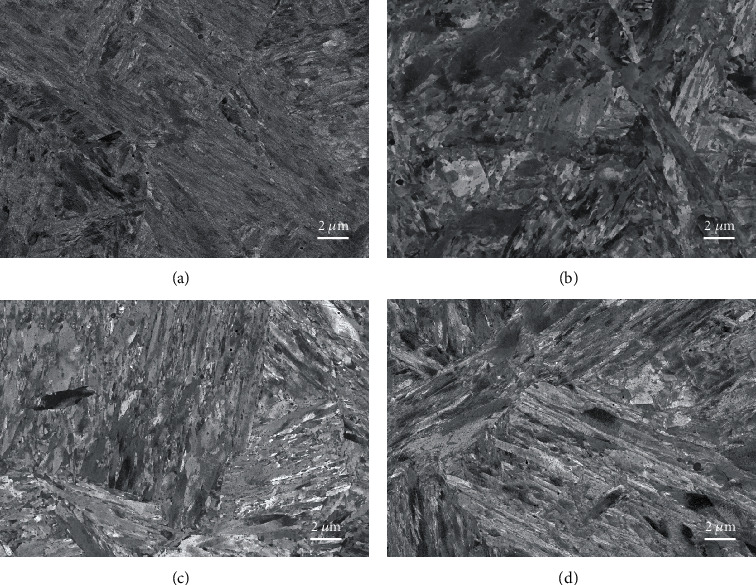
Microstructure overview for samples M12 (a), M16 (b), M23 (c), and M26 (d) using ECC in the SEM. The contrast is solely dependent on the crystal orientation and is uniform over the whole images.

The four samples were imaged using ECC, and the resulting images are displayed in Figure 3. Each image corresponds to a single-scan image for which the acquisition time was approximately 2 minutes. The image field of view and scale are of the same range as in Figure 2. It is, at first sight, very striking that the image contrast was uniform over the image field of view, since the electron beam diffusion is not influenced by the specimen thickness as for TEM, but, on the contrary, by the specimen density, the mean atomic number, and the crystal orientation [2]. From these low-magnification images, it appears clearly that the microstructure is mainly martensitic, but the presence of plate martensite is clearly visible for samples M16 and M23. This plate martensite was also observed on higher-magnification TEM images but not directly on the montage images in Figure 2. The SEM images are also not showing the delineation marks of the single images as seen in the montage images of Figure 2. It must be noted that the same montage methodology could also be used to produce large-field-of-view images with higher-magnification ECC images without seeing the image delimitation marks, as can be seen in the TEM montage image, since the contrast and brightness provided by ECCI are uniform over the flat surface of the specimen.

#### 3.1.2. Lath Structure

**Figure 4:**
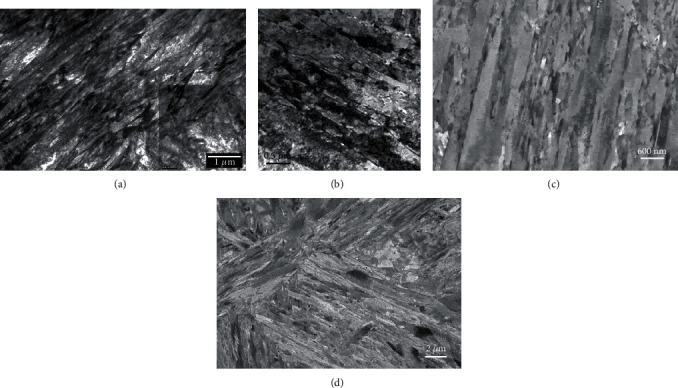
TEM-BF (a, b) and ECC images (c, d) of samples M23 (a, c) and M26 (b, d) showing the martensite lath structures observed in these alloys. TEM-BF image in (a) is a montage made of higher-magnification images to reduce the effect of specimen thickness on the image contrast.

A typical martensitic lath structure was developed in the four alloys as a result of the different tempering treatments as was shown in the TEM-BF images (Figures 4(a) and 4(b) for samples M23 and M26, respectively). Note that due to the thickness variations in the field of view of the image in Figure 4(a), a montage image had to be used to reduce this effect. The ECC images in Figures 4(c) and 4(d) corresponding to the same samples show the same level of detail, however with a uniform contrast. The lath structure has dimensions identical with those seen in the TEM images, and some dislocations can also be observed inside some of the darkest lath grains. This contrast is generally observed when the crystal is locally oriented to match a low index (hkl) Kikuchi line with the optic axis of the microscope. The consequent angle between the electron beam and the (hkl) crystal plane trace is then close to the Bragg angle for this plane. The local variation of the dislocation neighboring lattice plane orientation, due to the strain field around the dislocation core, induces a variation of the backscattering coefficient that lead the dislocation surrounding to appear brighter than the grain background [23, 40]. Note that the excitation condition and depth of the dislocation also play important roles in the dislocation visibility.

#### 3.1.3. Plate Martensite

**Figure 5:**
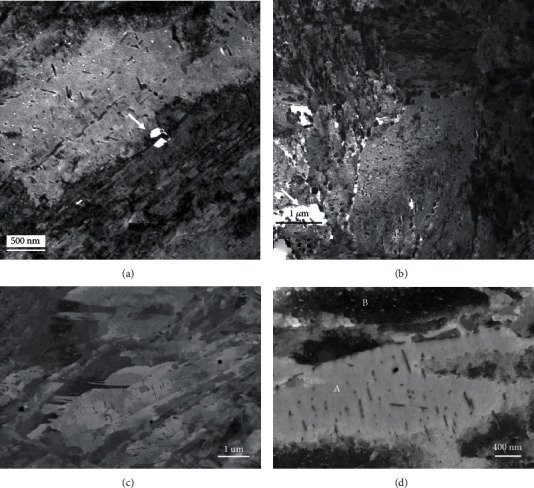
TEM-BF (a, b) and ECC (c, d) images of plate martensite in samples M16 (a, c) and M23 (b, d). Note: contrast inversion from carbides in different martensite grains in A and B in (d). White arrows in (a) and (b) indicate sample preparation artefacts.

It was noticed in the overview images obtained with SEM in Figure 3 that samples M16 and M23 contained a significant amount of plate martensite [41], compared to samples M12 and M26. Plate martensite is recognizable due to its smaller aspect ratio compared to lath martensite. This appears very clearly in the TEM images (Figures 5(a) and 5(b)) where the contrast inside the grains is somewhat uniform with very few deformation structures (e.g., twinning and bending) (Figure 4(b)) allowing the observation of carbides with high contrast. It must be noted, however, that some preparation artefacts, such as pits and overetched areas seen as bright spots in the TEM-BF images due to reduced thickness in these areas, degraded the image quality to some extent. The ECC images of the same two samples, shown in Figures 5(c) and 5(d), again provided a very uniform contrast and the same conclusions that were made with the TEM images regarding the carbide visibility inside the plate martensite grains. Deformation structures such as twinning (Figure 5(c)) or bending (Figure 5(d)) were also clearly observed inside those grains, and similar to Figures 4(c) and 4(d), single dislocations could be detected in dark grains. An interesting point in the SEM images, particularly in Figure 5(d), is the contrast of the carbides in plate and lath martensite as a function of the grain background. Brodusch and coworkers [30] reported that a contrast inversion can take place in polycrystalline alloys when the mean atomic number of the precipitate compound is very close to that of the matrix. In the present case, Fe_3_C carbides may appear darker or brighter than the grain matrix as shown in Figure 5(d) in A and B, respectively, depending on the orientation of the crystal versus the primary electron beam. This is a serious disadvantage of ECCI for characterizing carbides in these alloys and, to a greater extent, in steels in general. The methodology that was developed to address this problem will be explained in more detail in Section 3.1.6.

#### 3.1.4. Recovery Structure

**Figure 6:**
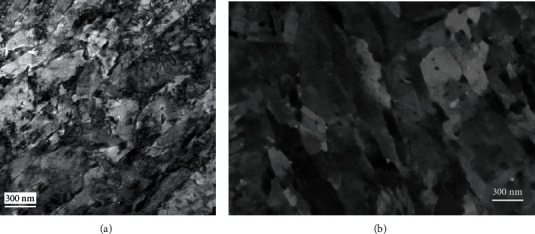
Recovery structures in sample M16 as imaged by TEM-BF (a) and ECC (b). The high angular resolution of ECCI (0.18° in the present case) allows imaging subgrains in the lath martensite.

Recovery structures are rearrangements of dislocations in martensite grains to form pseudo-cell structures or subgrain boundaries.  Figure 6(a) shows a TEM-BF image of sample M16 where subgrain boundaries can be identified, but their visibility is low compared to the SEM-ECC image as shown in Figure 6(b). On this latter image, the grain contrast is high and subgrains are clearly identified since the image angular resolution obtained with ECCI is relatively high. In fact, the high channeling contrast angular resolution is driven by the primary beam convergence angle, itself dependent on the working distance and objective aperture diameter, which was 8 mm and 50 *μ*m in this present study, leading to an approximate convergence semiangle of 0.18 degree.

#### 3.1.5. Twinning Structures

**Figure 7:**
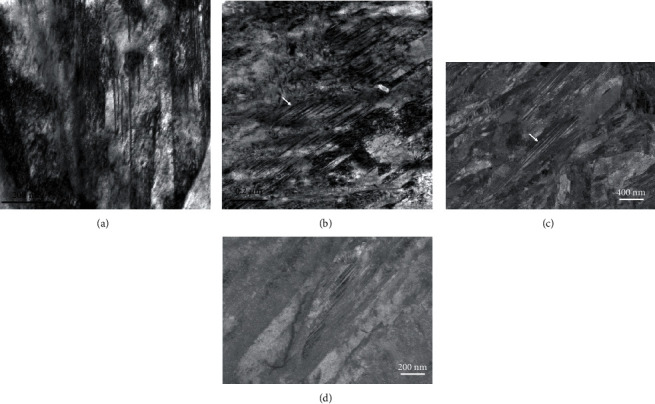
Twinning structures in samples M12 (a, c) and M26 (b, d) imaged with TEM-BF (a, b) and SEM-ECC (c, d). The twin size was between 2 and 10 nm in both TEM-BF and SEM-ECC images, but larger twins were observed by SEM-ECCI. The white arrows point to twinning structures as seen in BF-TEM and ECC images.

Typical twinning structures as seen in TEM-BF imaging are shown in Figures 7(a) and 7(b) for the quasi as-quenched samples M12 and M26. These twins are typical of as-quenched low-carbon steels [42, 43] and result from the shear accommodation inside the martensitic grains [44]. The twinning structure was confirmed by selected area diffraction (SAD). The corresponding SEM-ECC images displayed in Figures 7(c) and 7(d) show similar twins for samples M12 (Figure 7(c)) and M26 (Figure 7(d)). The observed twins are smaller than 10 nm as seen in either TEM or ECC images [43, 44]. However, some twins seen on ECC images can reach a few tens of nanometers, and they may be larger twins or, more likely, stacks of parallel nanosized twins. A few twins were identified in the two samples by TEM, mostly in plate or block martensite grains, but the ECC images suggested that twinning was more present in these two samples and was also occurring in few lath martensite grains [42].

#### 3.1.6. Carbides

**Figure 8:**
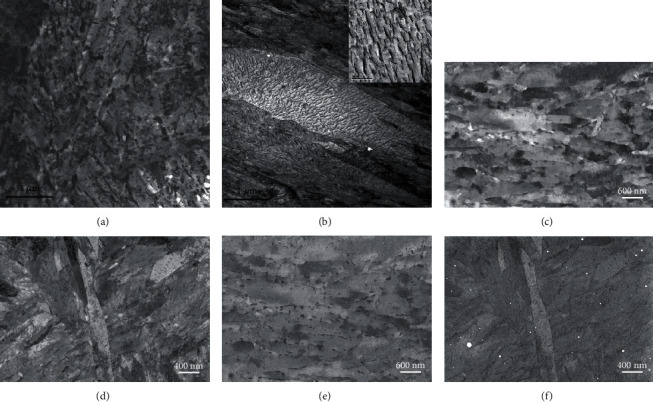
TEM-BF (a, b), SEM-ECC (c, d), and SE (e, f) images for samples M23 (a, c, e) and M12 (b, d, f). The inset in (b) shows a magnified image of the carbide structure in plate martensite. The SE images provide higher contrast between the carbide precipitates and the polycrystalline grain structure. Note that the bright particles in (f) are colloidal silica particles remaining on the surface after the final step of sample preparation.

When carbides are nanosized, TEM is the technique of choice to visualize them and examples of TEM-BF images from samples M23 and M12 are shown in Figures 8(a) and 8(b), respectively. The carbides in samples M16 and M23 were identified based on SAD patterns mainly as cementite, with a few precipitates being of the Fe_2_MoC type. In samples M12 and M26, all carbides were identified as transition *η*-carbides (orthorhombic, Fe_2_C) but some hexagonal *ε*-carbides were found in sample M26. Despite the diffraction contrast interfering with mass-thickness contrast in BF images, especially in Figure 8(a), it is clear from these images that the carbide size, shape, and spatial distribution are different in these two alloys. The carbides are smaller and more 2D-shaped in the quasi as-quenched sample (M12, Figure 8(b)) compared to the round-shaped larger carbides seen in the tempered sample (M23, Figure 8(a)). Also, precipitation seems to be more abundant inside plate martensite grains in sample M12.

SEM-ECC images of the same samples are shown in Figures 8(b) and 8(c) for M12 and M23, respectively, and the mixing of the composition and crystallographic contrast is similar to that observed in TEM-BF. As mentioned in Section 3.1.3, the modulation of the backscattering coefficient due to orientation contrast leads to coefficients larger or smaller than that of the carbides, which produces darker or brighter precipitates on the image, respectively. However, the different electronic structures from the native oxide layer at the surface of the steel matrix and from the carbides, reflected in the dielectric function, produce different secondary electron coefficients (see Section 2.7). Thus, this generates a high contrast between the carbides and the matrix, regardless of the grain orientation, making the carbides appear systematically darker than the matrix. In addition to providing enough contrast to identify the precipitates, SE imaging provides true surface imaging, since the depth of emission of SE is just a few nanometers, contrasting with the BSE diffusion volume, which is around 200 nm in iron at 10 kV. Thus, the image can be considered a slice of the specimen, and the volume fraction was taken as the surface fraction as the first approximation. Another advantage of this technique is that it allows the collection of the SE image along with the ECC image with one single scan, and consequently, it gives the ability to correlate carbide imaging with the crystallographic microstructure for each image. It must be mentioned, though, that because the SE signal is emitted from a very thin surface layer, carbon contamination can be a serious issue if the sample is not perfectly cleaned before the SEM characterization. In this work, an ozone cleaner was used to remove any organic species remaining after the polishing process.

The SE images corresponding to those in Figures 8(c) and 8(d) are shown in Figures 8(e) and 8(f), respectively. From these high-contrast images, the same conclusions made with the TEM images can be drawn, regarding the carbide size and shape. However, the localization of the carbides is clearer, and it appears that carbide precipitation is favored at the grain boundaries (Figure 8(e)) in tempered samples, compared to the quasi as-quenched samples, where precipitation is more uniform (Figure 8(f)). This high contrast allowed further segmentation of the images to gather quantitative information from the carbide morphology and distribution. This, combined with the large representativeness of the SEM analysis, enables excellent statistics, as will be shown in Section 3.2.

#### 3.1.7. Dislocations

Dislocation imaging in the SEM via ECCI is an established technique with defect imaging being predicted by Coates in 1967 [45] and reported experimentally on thin electron transparent specimens by Clarke in a scanning transmission electron microscope [46]. Later, Morin and coworkers [27] showed the first images of dislocations and stacking faults in bulk silicon with an accelerating voltage of 50 kV in a field-emission SEM. It was only in the late nineties that ECCI became a popular technique and is now used frequently to characterize dislocation structures in metals [24, 25, 47] and ceramics [26, 48, 49]. To image the various defects, it is important that the crystal is oriented in a quasi two-beam condition where only one set of planes provides strong diffraction following the Bragg relation [23]. In this condition, defects appear with darker intensity compared to the grain background intensity in TEM-BF while it is reversed when ECC is used, i.e., bright defect contrast on a dark background. ECC can be maximized for a particular location or grain in a sample by using controlled ECC where the necessary deviation parameter from the selected two-beam orientation is precisely adjusted with either electron backscatter electron diffraction (EBSD) [25, 48] or electron channeling patterns (ECP) [50–52]. However, these techniques were only applied to microstructures with large grain size, typically in the range of 5 to 30 *μ*m or larger, and are not applicable in our study since the microstructure is mainly composed of submicrometer lath martensite. Instead, since the SEM provides a very large number of grains of varying orientations, dark grains were targeted since a low BSE intensity is evidence of a high channeling condition, indicating that the Bragg condition is more or less satisfied. In addition, small-angle tilting and rotation can be applied to adjust the two-beam condition to optimize the dislocation contrast [48].

**Figure 9:**
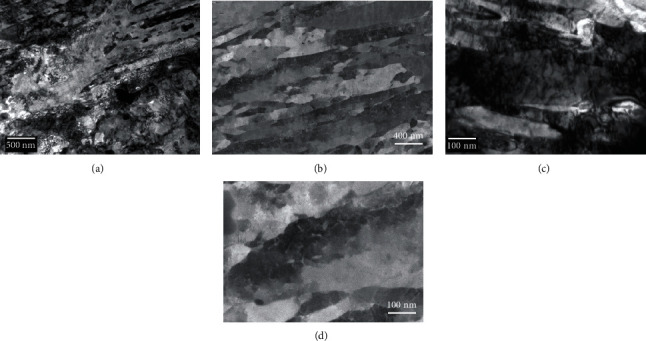
TEM-BF (a, c) and SEM-ECC (b, d) images of sample M16 showing dislocation arrangement in the microstructure.

A comparison of the imaging contrast achieved with TEM-BF and SEM-ECC is shown in Figure 9 for the tempered sample M16. The TEM-BF image in Figure 9(a) shows packets of dislocations spread over lath martensite grains with a higher concentration at the grain boundaries. A higher magnification is shown in Figure 9(c) where single and tangled dislocations are resolved. However, due to the large number of defects, it is difficult to resolve individual dislocations in the darkest area. This is particularly true when the foil thickness is large because the TEM image is a projection of all defects located in the foil volume. The SEM-ECC images shown in Figures 9(b) and 9(d) correspond to the same M16 sample with similar scales as in Figures 9(a) and 9(c). The dislocations are clearly visible in dark areas (i.e., close to two-beam Bragg condition) but also in slightly lighter areas with increasing deviation parameters from the exact Bragg angle [53]. Single dislocations were clearly resolved, and dislocation tangles were observed as in the TEM images. However, the tangles seem less dense in the SEM-ECC images, which might be a result of a smaller depth of emission of the BSE signal carrying the diffraction information. This depth depends on the diffraction condition and on the material and is a multiple of the extinction distance for this condition [23]. Zaefferer and Elhami estimated the depth resolution to be a few tens of nanometers in Fe with the (111) reflection at 20 kV [23]. Berger and Niedrig [54] have shown that only BSEs with less than 20% energy loss were contributing to the ECP, but some authors assume a much smaller energy loss for these contributing electrons [55, 56]. Thus, Monte Carlo modelling can be used to monitor the trajectories of these low-loss backscattered electrons and allows the evaluation of the depth resolution. A simulation with the Casino software [57] with 5 × 10^6^ electrons and an energy loss of 10 and 20% (supplementary material, Figure S3) with an accelerating voltage of 10 kV was computed. The emission depth was then evaluated to approximately 30 nm and 50 nm for energy loss of 10% and 20%, respectively. It must be mentioned here that diffraction effects were not accounted for in this simulation, but the depth resolution was consistent with the calculations of Zaefferer and must be regarded as an upper estimation. This shows clearly that the emission volume contributing to the image might be smaller using SEM-ECCI compared to conventional thicknesses used in TEM experiments, i.e., more or less 100 nm. Thus, less dislocations might be “seen” by the BSEs along the emission volume, compared to the TEM thickness, when a high density of dislocations is observed, reducing the diffuse background of the ECC image and rendering them more visible than with TEM in such samples.

### 3.2. Quantitative Analysis

#### 3.2.1. Width of Martensite Laths

**Figure 10:**
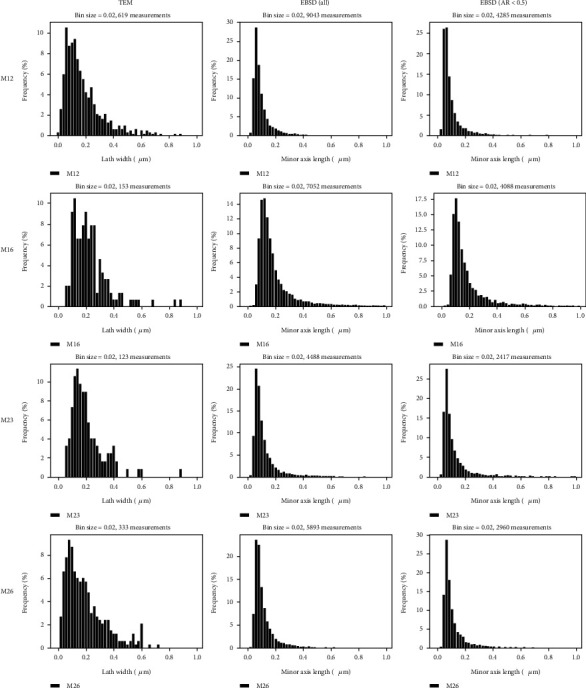
Martensite lath width distribution for samples M12, M16, M23, and M26 obtained with TEM manual measurement and EBSD automatic grain detection based on grain boundaries greater than 15°. The right column shows the distribution from EBSD data with grains having an aspect ratio (AR) smaller than 0.5, i.e., elongated grains as expected from lath structures.

In most cases, TEM studies require manual measurement of martensite lath grain dimensions from TEM images, which is time consuming and subjective in the selection of which object to be measured. The TEM martensite lath width measurements obtained from samples M12, M16, M23, and M26 are displayed in Figure 10 (left column). While the martensite lath width distribution was quite similar for the four samples, with size ranging from 20 to 600/800 nm, one can note slight differences. The distribution maximum, which was about 150 nm for the lower-hardness samples M16 and M23, shows a pronounced shift towards smaller lath width at around 100 nm for the hardest samples M12 and M26. Also, the distribution spread around the maximum was larger for samples M12 and M26 compared to the other two samples. However, the lack of measurements to produce these histograms might reduce the accuracy of these findings. Also, the fact that only the diffraction contrast from the BF images was considered to identify the lath without measuring the actual angle between them might bring some inaccuracies in the corresponding grain size distributions.

The quality (band contrast) and grain maps obtained by EBSD are given in Figure S4 for the four samples. At first sight, the martensite grain dimensions seem to be quite similar from one sample to another. However, the size distributions extracted from each map and shown in Figure 10 draw a slightly different scenario. In the column “EBSD (all)” (middle column), the distribution of the grain's width is given for the four samples. Here, the width corresponds to the small (minor) axis of each detected grain on the maps. Since most of the grains are martensite laths, the distribution can be considered representative of the lath width distribution and hence can be compared with TEM results. The width distribution measured by EBSD shows a similar trend as what was observed from the TEM measurements; i.e., the martensite lath width is smaller for samples M12 and M26. However, sample M23 shows also smaller grain width and only sample M16 has larger grains. Specifically, the distribution maximum was found to be around 60-80 nm for samples M12, M23, and M26 while it is found to be around 100-120 nm for sample M16. For all samples, the distribution ranged from around 20 nm to 600/800 nm which is of the same order of magnitude as reported from TEM measurements. However, the EBSD histograms are smoother in the large width portion of the graphs, since a higher fraction of larger grains could be intercepted with the field of view used to record the EBSD maps. In addition, it must be inferred that the EBSD measurements had more statistical significance since the distributions were computed with several thousand grain measurements compared to a few hundred for the TEM analysis.

It must be inferred, however, that due to the limited angular resolution of the EBSD analysis with the presence of the magnetic field, as explained in Section 2.5, the differentiation of single lath grains, even with threshold angles of 2° or 3°, was very unlikely. The martensite lath as described here might be considered lath packets or blocks, which, combined to the TEM inaccuracies due to not considering the lath angles as well, makes the comparison somewhat less accurate, although still informative when comparing between different samples if the same procedure is being used.

**Figure 11:**
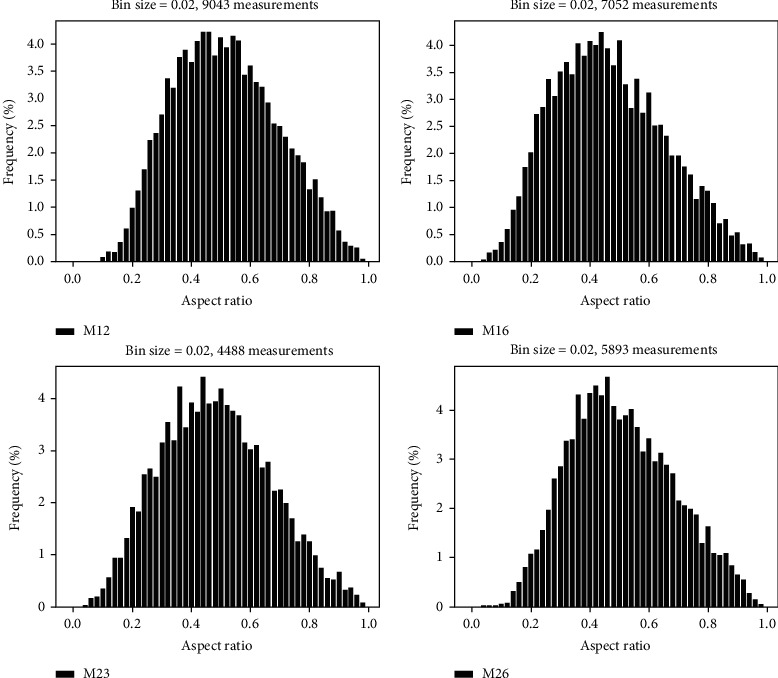
Martensite grain aspect ratio (AR) as the ratio between the minor and major axes of the grain for samples M12, M16, M23, and M26.

Since the EBSD analysis comprised all martensite grains, lath, and plates, the width size distribution of the grains was replotted with only grains having an aspect ratio (AR, Figure 11), defined as the small axis over the long axis lengths, smaller than 0.5. This value of AR was chosen to account only for lath grains in the distribution since plate martensite grains have generally smaller AR values as seen from ECC images. The distributions for the four samples are shown in Figure 10 as “EBSD (AR < 0.5)” on the right column. The distribution spread hence obtained was very similar to that obtained with all grains of the maps, which was expected, since the fraction of plate martensite observed in ECC images was quite small. Also, it might be noted here that since the EBSD grain distribution is solely based on the pixel absolute orientations, it is considered more accurate in defining grains than the visual inspection of TEM-BF images where the contrast is complex and might be similar for different grain orientations.

It must be mentioned also that the aspect ratio distribution obtained by EBSD grain measurements informs on the grain dimensions, as seen from Figure 11. Therefore, although the four histograms have similar shapes, the distributions have their maximum at different ratios, i.e., around 0.4 for M16 and M26 and 0.5-0.55 for samples M12 and M23. Also, a small shoulder is seen at around 0.2-0.25, which seems to be more pronounced for samples M12 and M16 compared to the two other samples. This means that a larger fraction of thin martensite needles is present in alloys M12 and M16 compared to samples M23 and M26, underlining a difference in the martensitic microstructure, which apparently is too slight to affect the strength and hardness, which are all similar.

#### 3.2.2. Carbide Characterization by Deep Learning Segmentation of SEM Images

**Figure 12:**
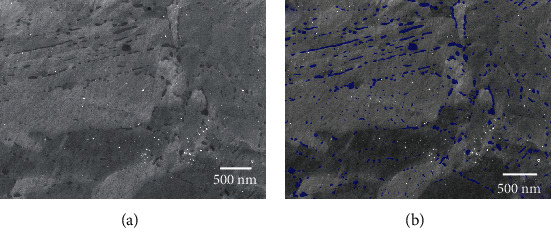
(a) Original SE and (b) segmented images from sample M23. Segmentation was done by applying a deep learning model trained with a set of experimental images cropped to a quarter of the original image area. Segmented particles are colored in blue in image (b).

*(1) Size and Shape Distributions*. The precipitation of carbide nanoparticles was studied for the four samples by applying deep learning segmentation to extract each single carbide from every secondary electron image recorded simultaneously to the BSE images in the SEM. An example of the typical segmentation resulting from applying deep learning is given in Figure 12, where the original SE image is shown in Figure 12(a) and the segmented image in Figure 12(b). Segmented carbides were colored in blue to highlight them over the grey level image. The segmentation is highly precise, and either large or nanometer-range particles were successfully identified by the model. The bright clusters of pixels represent remnants of colloidal silica particles from the final polishing step and, as expected for a network trained to recognize carbides only, none of them was included in the segmentation results. Another advantage of using a deep learning model lies in differentiating carbides from other surface features, such as shallow scratches resulting from the final polishing step with silica nanoparticles. These lines, seen in Figure 12(a), have very similar grey levels compared to the small carbide particles. Nonetheless, the model clearly made the difference between these two types of features, and again, none of the scratches were considered carbides in the final segmented images.

**Figure 13:**
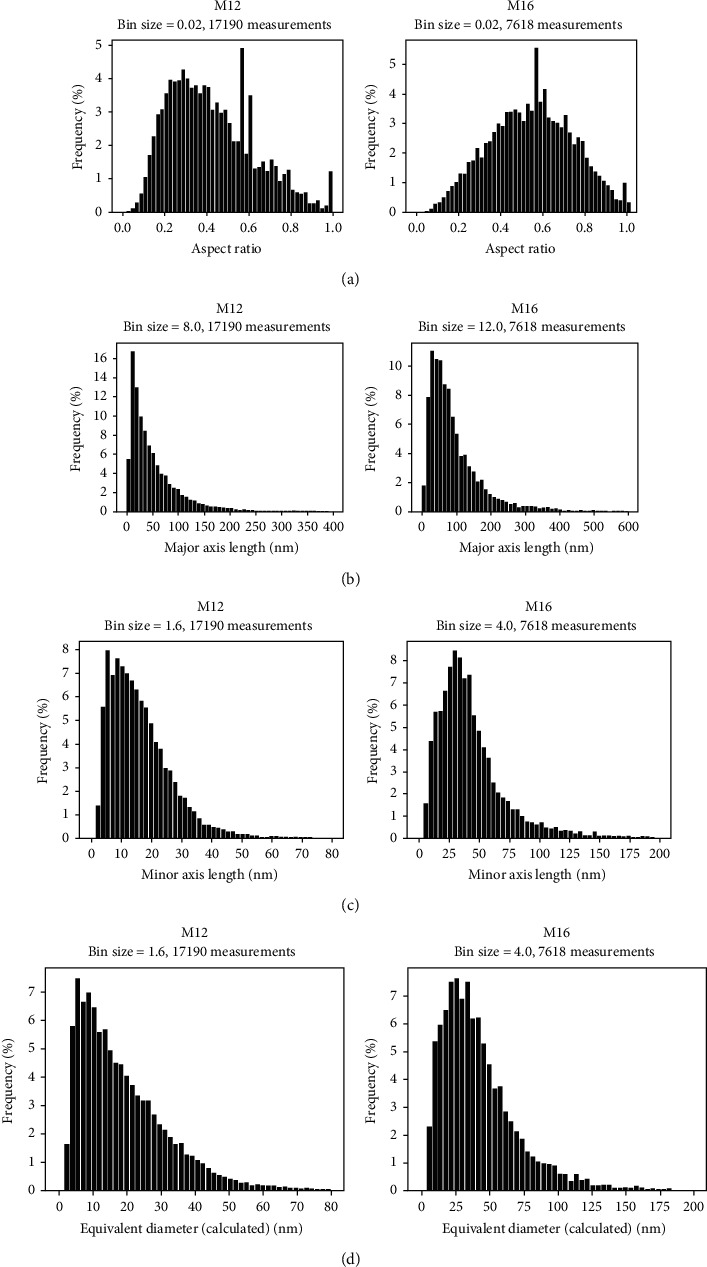
Carbide aspect ratio (a) and size distributions for samples M12 and M16 obtained by deep learning processing of SE images recorded with the SEM. The size was characterized as the long (major) (b) and small (minor) (c) axes of each detected particle as well as a calculated equivalent circle diameter (d).

The segmentation and processing of the detected particles with the Dragonfly software enabled the production of size distribution histograms, which are presented in Figure 13 for samples M12 and M16 by processing a large set of images to account for the representativeness of the SEM analysis. The complete set of histograms for all samples, including samples M23 and M26, is presented in Figure S6, and the histograms obtained from manual measurements on TEM images are given in Figure S5. The carbide geometry was investigated using four metrics, namely, the aspect ratio (Figure S6(a)), the major (Figure S6(b)) and minor (Figure S6(c)) axis length of a fitted ellipse on the particle, and the equivalent diameter (Figure S6(d)) calculated considering a perfect disk of the same area as the particle.

Clearly, the carbide aspect ratio maximum for sample M12 is dramatically smaller than for sample M16, namely, 0.25-0.3 and 0.55-0.6, respectively (an aspect ratio of 1 stands for a circular particle). This confirms the visual inspection of the SE and BSE images where roundish and elongated particles were observed in samples M16 and M23 while the carbides looked more like thin platelets in samples M16 and M26 (Figure 8). The most probable carbide equivalent diameter (*d*) for sample M12 is smaller, around 10 nm in average, than for sample M16, around 30 nm. However, sample M12 shows a narrower size spread (2 to 70 nm) than sample M16 (5 to 150 nm). So, clearly, the carbides in sample M16 are larger than those in sample M12, and this was confirmed by the minor and major fitted ellipse axis distribution, where not only the width of the carbides is reduced but also their length. This highlights the effect of increased tempering time from M12 to M16, which increased the amount of carbon diffusion out of the martensite grains to coarsen carbide particles, which increases the amount of carbide formed. This was confirmed by calculating the carbide area fraction over the total area of the processed images, and the average fractions are given in Table 3 for the two samples. The carbide fraction doubles from 1.9 ± 0.5 area % in sample M12 to 3.8 ± 0.3 area % in sample M16 confirming, since both samples have nearly the same carbon content, the increased amount of transformation from martensite to form carbide precipitates with longer tempering times.

It must be noted at this stage that this comparison is based on images obtained from two-dimensional sections (TEM and SEM) which may not fully reproduce the exact particle size that would be measured based on a three-dimensional characterization. This comparison is nonetheless useful to evaluate the SEM capability to provide similar particle size as those measured by TEM.

#### 3.2.3. Retained Austenite

**Figure 14:**
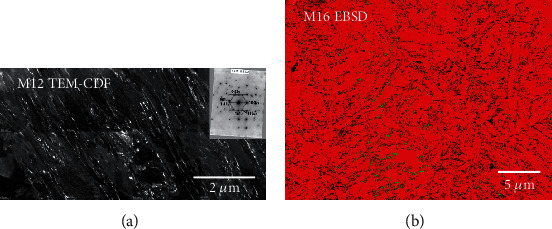
TEM-CDF image for sample M12 (a) and zoomed view of the EBSD map for sample M16 (b) (see Figure S7 in the supplementary material file for the complete EBSD map). Red color in the EBSD map stands for martensite and green for austenite. The TEM image is a montage of eight high-resolution CDF images. Black pixels correspond to nonindexed pixels. The inset image shows an example of a SAD pattern describing the relationship between retained austenite and martensite in sample M12.

Here, TEM imaging using centered dark field (CDF) [3, 58] was used to investigate the four samples of the study. Retained austenite thin films of the order of 50 to 200 nm were observed between martensite lath grains in samples M12 and M26 as shown in Figure 14 for sample M12. The selected area diffraction pattern shown in the inset of the CDF image describes the relationship between the *α*′ martensite laths and the *γ* retained austenite. However, it was not possible to quantify the volume fraction since the image contrast in CDF imaging is complicated and difficult to interpret in this regard. Moreover, X-ray diffraction did not detect retained austenite due to its low volume content. Note that CDF imaging did not permit the identification of any retained austenite in tempered samples M16 and M23.

Since it provides phase differentiation [59], EBSD can identify retained austenite in martensite as the two phases differ from each other mainly by their crystal system. The bct martensite is close to the bcc structure, the latter being differentiated from the fcc lattice of austenite by the Hough transform-based indexing procedure of EBSD. Fundamentally limited by the intrinsic spatial resolution of the diffracted backscattered electrons, which is just a few tens of nanometers [60, 61], this technique only applies to quite thick films of retained austenite. Phase differentiation was applied to the four samples, and the resultant phase maps are given in Figure S7 (supplementary material file) with total indexing rates ranging from 85.8% to 94.6%. On these maps, austenite is colored in green and martensite in red. At first inspection, austenite was detected in all samples, mainly at the lath boundaries, but some pixels inside martensite can be observed as well. These pixels might be considered having misindexed pixels since retained austenite was not expected inside martensite. In fact, isolated single pixels have no meaning and must be considered noise in the map. However, some packets of austenite pixels were clearly localized at the lath boundaries indicating the presence of austenite thin films as reported by TEM-CDF. A closer look at the orientation data of the maps (not shown) indicates that the clusters present identical orientation which confirms that these may not be noise clusters. This also shows that most of these clusters might belong to a single primary austenite grain since they retained the same orientation after the martensitic transformation. More importantly, austenite is detected in all samples but is not uniformly distributed over the surface of the maps. For example, some areas of the map clearly showed a larger concentration in retained austenite at lath boundaries in sample M16 as reported in Figure 14. This was not expected since TEM-CDF investigations did not reveal the presence of retained austenite in tempered samples M16 and M23. It is assumed, here, that this might be a combined effect of the nonuniform distribution revealed by EBSD with the highly localized aspect of TEM characterization, as previously underlined. It must be kept in mind, also, that strain-induced austenite might have been a result of preparation and holding of the foils. The area fractions of austenite in each sample were extracted from the EBSD data and are given in Table 4. It can be concluded from these results that the austenite content is in the same range for the four samples and shows that tempering seemed to have a very limited effect on retained austenite fraction. It must be inferred that the indexing rate might affect the calculated phase relative fractions, especially when retained austenite exists in the form of nanometer-scale crystallites in fine martensitic structures. However, we consider that with similar indexing rates and microstructures, the area fractions extracted from the EBSD data can still be used to compare between samples of different origins.

## 4. Conclusion

In this work, TEM and SEM techniques were evaluated and compared in characterizing high-strength steel microstructures in quasi as-quenched and tempered conditions. Both techniques were used to provide detailed characterization, qualitative and quantitative for certain aspects. It was found that, although having a slightly inferior spatial resolution compared to transmission techniques, FE-SEM data can offer comparable capability as TEM regarding grain microstructure and second-phase precipitation. The findings are summarized as follows:

1. The microstructure of high-strength steels as imaged by SEM-ECCI provides a fast and uniform overview of the typical features of the materials, i.e., martensite laths and plate martensite
2. At higher magnification, in addition to the lath and plate martensite, recovery as well as dislocations and twinning structures was assessed on a large scale
3. Martensite grains were successfully analyzed using automated EBSD and postprocessing to provide quantitative grain size distributions based on thousands of grains allowing the extraction of specific grain distributions based on the grain aspect ratio
4. Retained austenite content was measured using EBSD and showed a similar surface content in the four samples investigated which completed previous TEM conclusions that no retained austenite was present in the tempered samples. It was also shown that the austenite surface distribution was not uniform for all samples
5. Carbide precipitation was clearly characterized using secondary electron imaging. It provided high contrast between carbides and the steel matrix and allowed the use of deep learning models to precisely segment images and retrieve carbide size and shape information automatically. Further size distribution histograms were produced from thousands of measurements and were comparable to the TEM measurements. However, it has to be kept in mind that the time allocated to manual segmentation of the images for feeding the deep learning model remains over all the main limitation of the technique. Using a larger set of images for training the model will certainly bring higher accuracy and representativeness compared to what would be obtained with fewer images and smaller manual segmentation time

In conclusion, it was found that FE-SEM can provide data that are similar in nature and quality to TEM regarding grain microstructure and second-phase precipitation, albeit with a slightly lower spatial resolution. This finding represents a breakthrough in the analytical capability of FE-SEM. The advantage of SEM is that it offers the possibility to observe a much larger proportion of the sample than TEM, thus generating data that are more reliably representative of the bulk material. With analytical capability that is comparable to TEM and the benefit of data that are more representative and more readily obtained, FE-SEM is more relevant in assessing the material mechanical properties. It might be kept in mind also that SEM offers the possibility to characterize alloy microstructures more deeply by using EBSD and ECC techniques to gather information of the primary austenite grains or martensite lath/retained austenite orientation relationships.

Even if FE-SEM can be substituted to assess the microstructure and precipitation mechanism, TEM remains the technique of choice for atomic resolution and its ability to provide crystallographic information from nanometer-scale features is highly advantageous.

## Figures and Tables

**Table 1:** Elemental composition of alloys A2 and A6.

	AISI	Elemental alloy composition in weight percentages (balance is iron)										
		C	Mn	P	S	Si	Cu	Ni	Cr	Mo	Al	V
A2	5140SKV	0.400	0.750	0.011	0.018	0.210	0.140	0.040	0.760	0.010	0.001	0.023
A6	4135MLV	0.350	0.900	0.010	0.015	0.250	0.060	0.040	0.950	0.160	0.001	0.020

**Table 2:** Heat treatment history and subsequent Rockwell hardness for the four specimens M12, M16, M23, and M26.

	Rockwell hardness (HRC)	Heat treatment history					
		Hardening	Temper 1	Temper 2	Rehardening	Temper 3	Temper 4
M12	52.8	843°C, 0.8 h	93°C, 2 h	468°C, 2 h	843°C, 0.9 h	163°C, 2 h	—
M16	34.8	843°C, 0.8 h	93°C, 2 h	496°C, 3 h	—	516°C, 3 h	538°C, 2 h
M23	36.6	857°C, 1 h	93°C, 2 h	482°C, 4 h	—	522°C, 2 h	546°C, 2 h
M26	51.5	857°C, 1 h	93°C, 2 h	—	—	—	—

**Table 3:** Carbide area fraction obtained from SE images segmented using deep learning and the corresponding standard deviations.

Sample	Carbide area fraction	Standard deviation
M12	0.019	0.005
M16	0.038	0.003
M23	0.049	0.009
M26	0.014	0.002

**Table 4:** Area percentages for martensite and retained austenite determined by EBSD.

Samples	Area percentage	
	Martensite	Austenite
M12	95.9	4.1
M16	95.4	4.6
M23	96.6	3.4
M26	96.5	3.5

## Data Availability

Due to confidentiality concerns, these data cannot be shared, except for the editor if required.

## Supplementary Materials

Supplementary Materials Supplementary Materials Figure S1: example of manual measurement along a line perpendicular to the lath long axis on a TEM-BF image. Figure S2: secondary electron (SE) image and corresponding X-ray maps for iron and carbon confirming that the dark precipitates on the SE image are carbides. The intensity in the X-ray maps was expressed as the *f*-ratio, i.e., the element's intensity normalized by the total intensity (Horny 2010). Figure S3: depth of emission of backscattered electrons in Fe with *E*_0_ = 10 kV with (a) 10% and (b) 20% energy loss obtained by Monte Carlo modelling with 5 × 10^6^ electrons. Figure S4: band contrast and grain maps obtained by EBSD on samples M12, M16, M23, and M26. The grains were detected using the Bruker Esprit software with a minimum of 10 pixels and a minimum boundary angle of 15°. Black pixels correspond to nonindexed pixels in the EBSD maps. Figure S5: carbide size distributions for samples M12 and M16 obtained by manual measurements on TEM-BF images. The size was characterized as the long (major) (b, d) and small (minor) (c, e) axis of each detected particle in lath (a–c) and plate (d, e) martensite. Figure S6: carbide aspect ratio and size distributions for samples M12, M16, M23, and M26 obtained by deep learning processing of SE images recorded with the SEM. The size was characterized as the long (major) (b) and small (minor) (c) axes of each detected particle as well as a calculated equivalent circle diameter (d). Figure S7: EBSD phase maps for samples M12, M16, M23, and M26. Red color stands for martensite and green for austenite. Black pixels correspond to nonindexed pixels.
