# Supplementary material for: Scanning Electron Microscopy versus Transmission Electron Microscopy for Material Characterization: A Comparative Study on High-Strength Steels
Source: Scanning. 2021 May 4;2021:5511618. doi: 10.1155/2021/5511618 (PMC8112914; doi:10.1155/2021/5511618)
Supplement: Supplementary Materials — Figure S1: example of manual measurement along a line perpendicular to the lath long axis on a TEM-BF image. Figure S2: secondary electron (SE) image and corresponding X-ray maps for iron and carbon confirming that the dark precipitates on the SE image are carbides. The intensity in the X-ray maps was expressed as the f-ratio, i.e., the element's intensity normalized by the total intensity (Horny 2010). Figure S3: depth of emission of backscattered electrons in Fe with E0 = 10 kV with (a) 10% and (b) 20% energy loss obtained by Monte Carlo modelling with 5 × 10^6^ electrons. Figure S4: band contrast and grain maps obtained by EBSD on samples M12, M16, M23, and M26. The grains were detected using the Bruker Esprit software with a minimum of 10 pixels and a minimum boundary angle of 15°. Black pixels correspond to nonindexed pixels in the EBSD maps. Figure S5: carbide size distributions for samples M12 and M16 obtained by manual measurements on TEM-BF images. The size was characterized as the long (major) (b, d) and small (minor) (c, e) axis of each detected particle in lath (a–c) and plate (d, e) martensite. Figure S6: carbide aspect ratio and size distributions for samples M12, M16, M23, and M26 obtained by deep learning processing of SE images recorded with the SEM. The size was characterized as the long (major) (b) and small (minor) (c) axes of each detected particle as well as a calculated equivalent circle diameter (d). Figure S7: EBSD phase maps for samples M12, M16, M23, and M26. Red color stands for martensite and green for austenite. Black pixels correspond to nonindexed pixels. [file 5511618.f1.pdf]

Supplementary material for the manuscript entitled: "Scanning Electron Microscopy versus Transmission Electron Microscopy for Materials Characterization: A Comparative Study on High Strength Steels"

Nicolas Brodusch[1], Salim V. Brahimi[2], Evelin Barbosa De Melo2, Jun Song[2], Stephen Yue[2], Nicolas Piché[3] and Raynald Gauvin[1].

[1]McGill Electron Microscopy Research Group, Department of Mining and Materials Engineering, McGill University, Montréal, Québec, H3A 0C5, Canada

[2]McGill Hydrogen Embrittlement Facility, Department of Mining and Materials Engineering, McGill University, Montréal, Québec, H3A 0C5, Canada

[3]Object Research Systems, 760 St-Paul West, Suite 101, Montreal, Quebec, Canada H3C 1M4

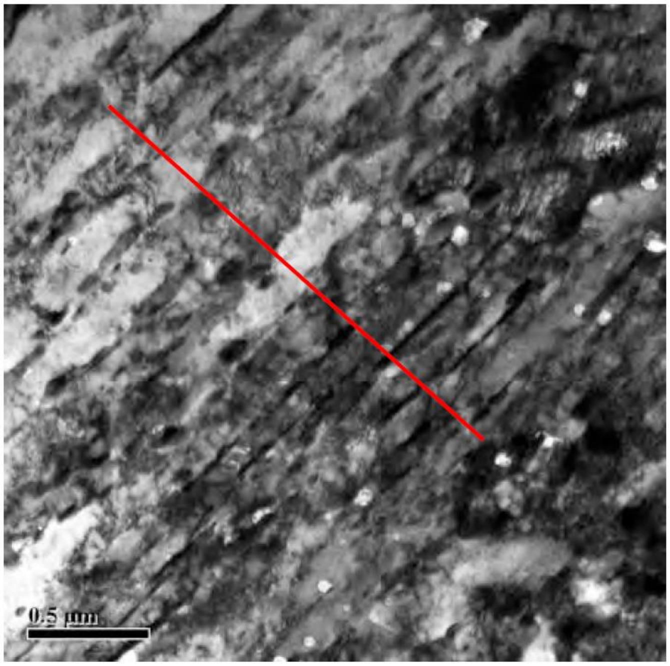

Figure S1: Example of manual measurement along a line perpendicular to the lath long axis on a TEM-BF image.

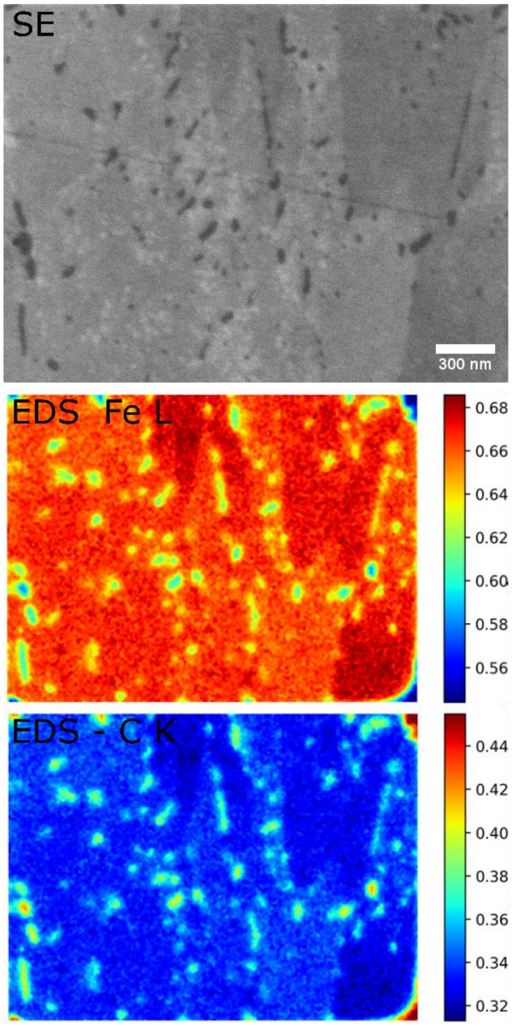

Figure S2 : Secondary electrons (SE) image and corresponding x-ray maps for iron and carbon confirming the dark precipitates on the SE image are carbides. The intensity in the x-ray maps was expressed as the f-ratio, i.e. the element's intensity normalized by the total intensity {Horny2010}.

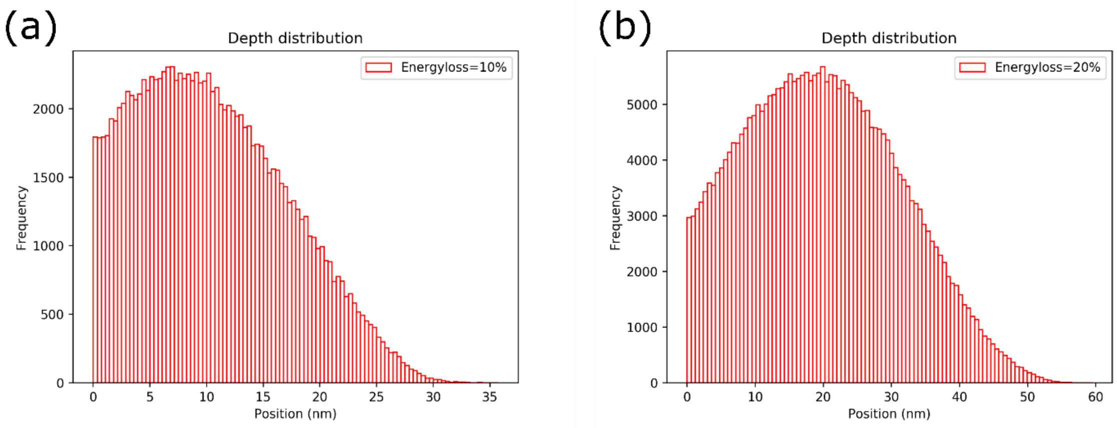

Figure S3: Depth of emission of backscattered electrons in Fe with E_0 = 10 kV with (a) 10 % and (b) 20 % energy-loss obtained by Monte Carlo modelling with 5 × 10^6 electrons.

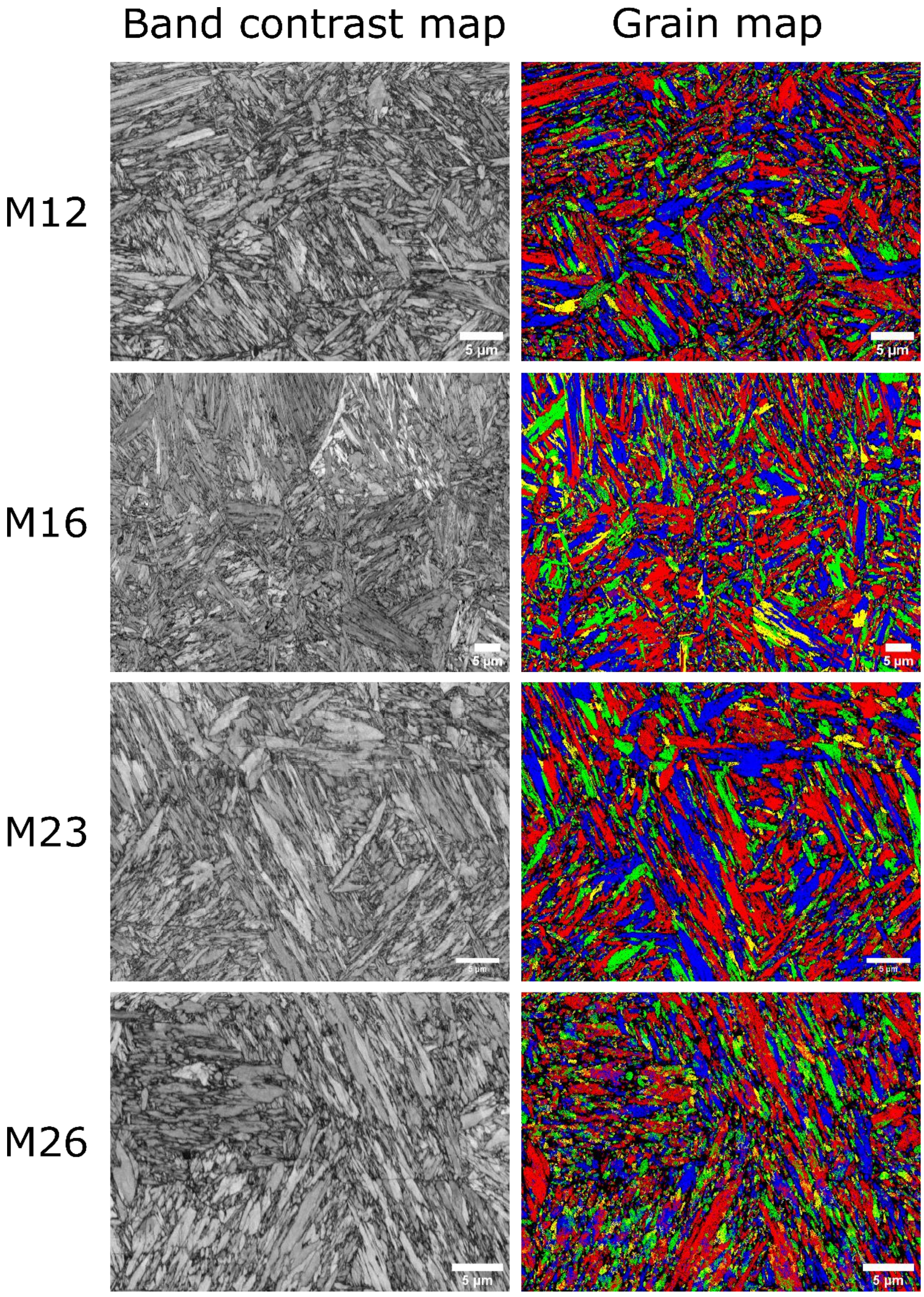

Figure S4: Band contrast and grain maps obtained by EBSD on samples M12, M16, M23 and M26. The grains were detected using the Bruker Esprit software with a minimum of 10 pixels and a minimum boundary angle of 15°. Black pixels correspond to non-indexed pixels in the EBSD maps.

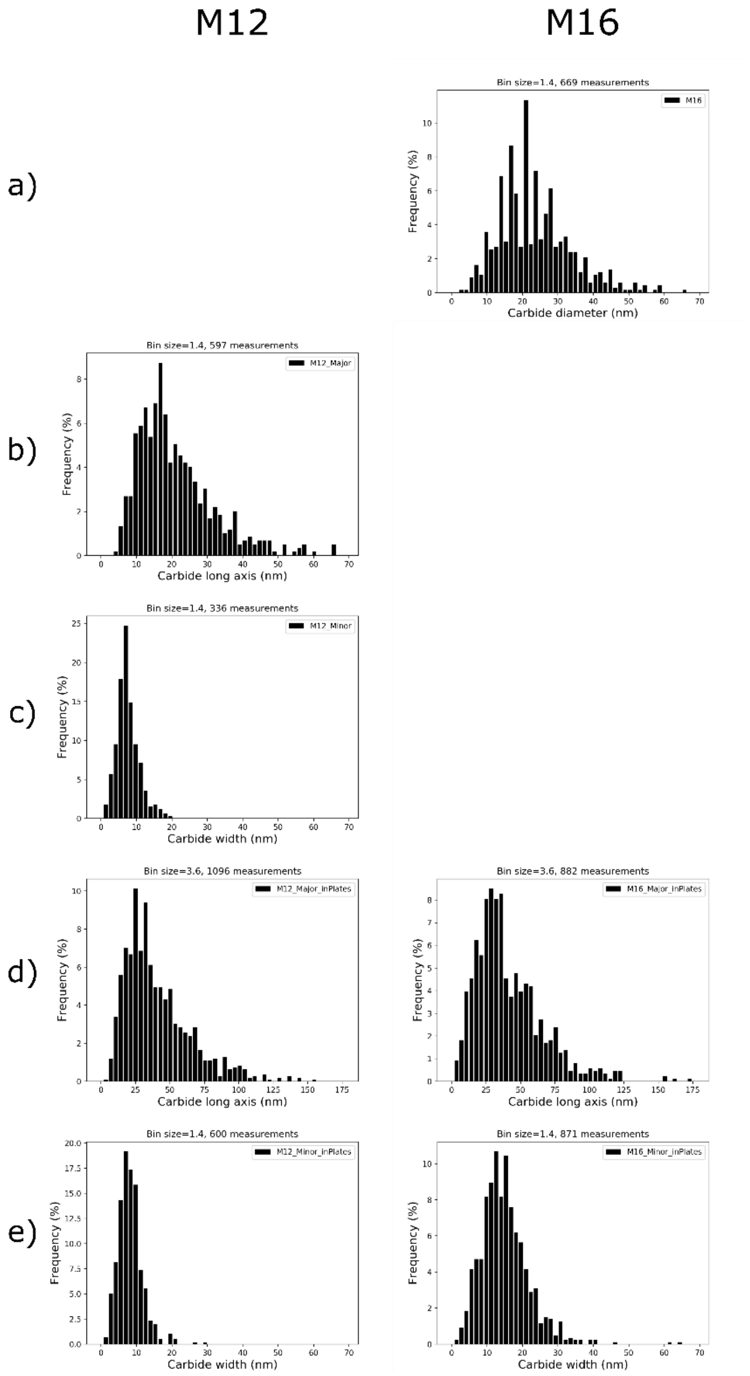

Figure S5: Carbide size distributions for samples M12 and M16 obtained by manual measurements on TEM-BF images. The size was characterized as the long (major) (b, d) and small (minor) (c, e) axis of each detected particle in lath (a-c) and plate (d,e) martensite.

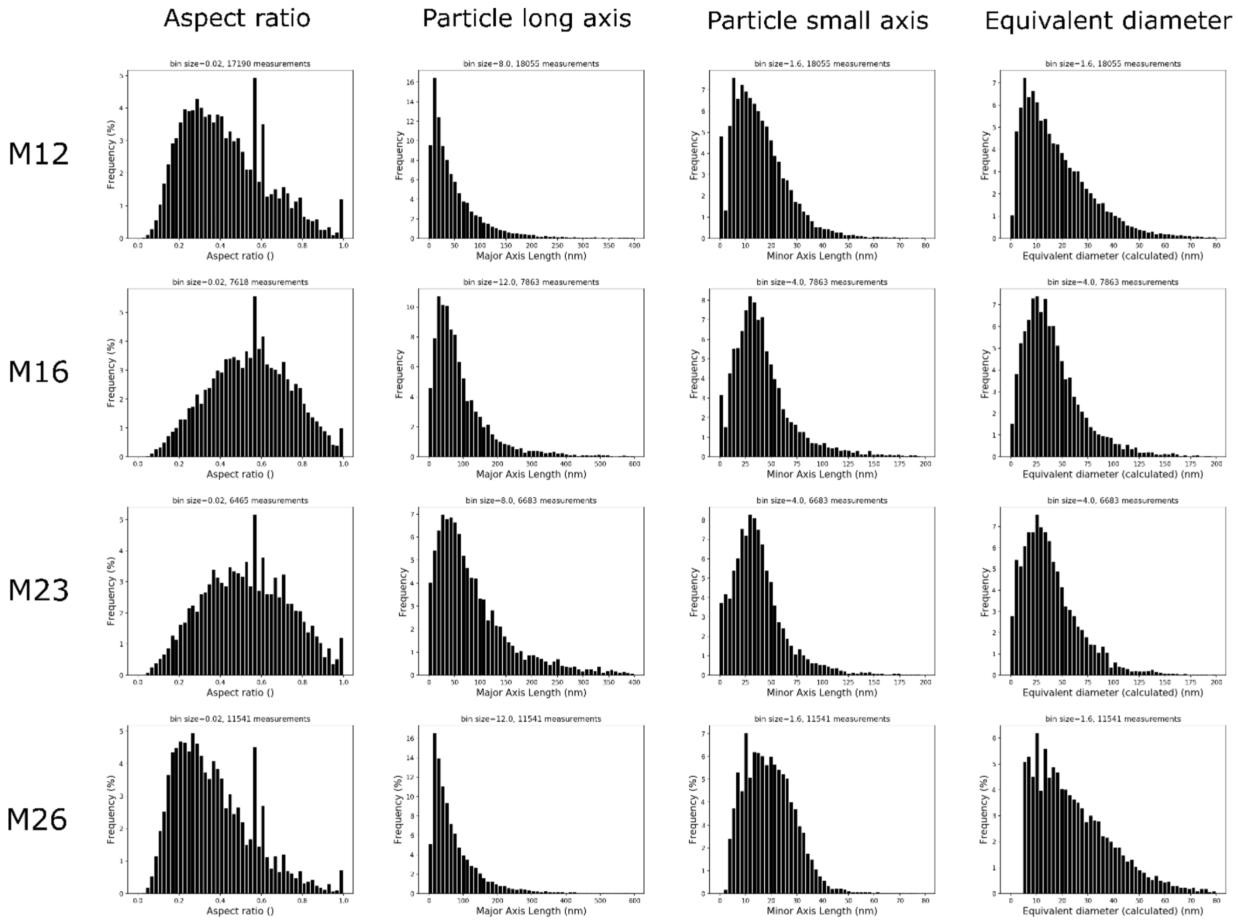

Figure S6 : Carbide aspect ratio, size distributions for samples M12, M16, M23 and M26 obtained by deep learning processing of SE images recorded with the SEM. The size was characterized as the long (major) (b) and small (minor) (c) axis of each detected particle as well as a calculated equivalent circle diameter (d).

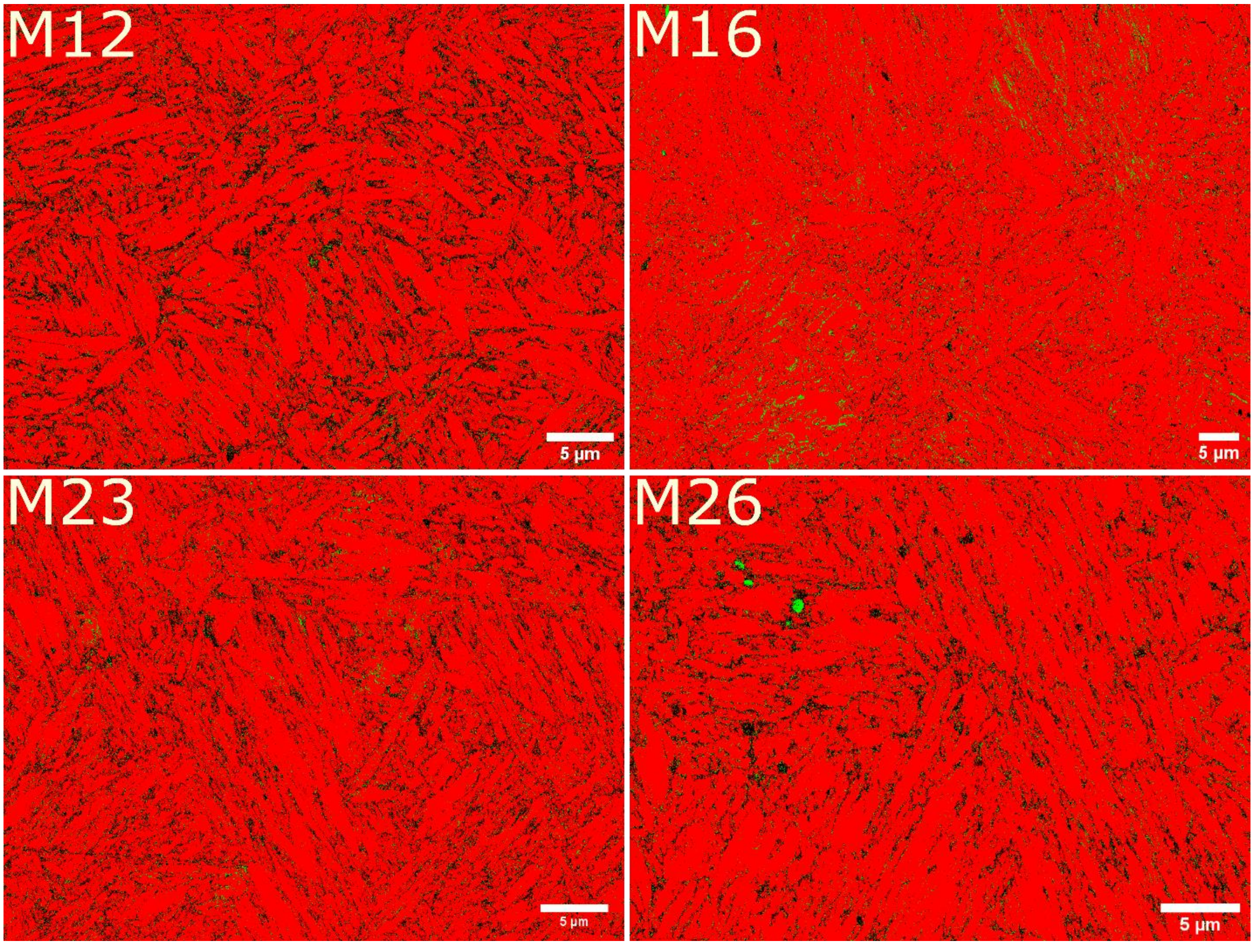

Figure S7: EBSD phase maps for samples M12, M16, M23 and M26. Red color stands for martensite and green for austenite. Black pixels correspond to non-indexed pixels.
